# A Palette of Cytokines to Measure Anti-Tumor Efficacy of T Cell-Based Therapeutics

**DOI:** 10.3390/cancers13040821

**Published:** 2021-02-16

**Authors:** Prathyaya Ramesh, Rohan Shivde, Dinesh Jaishankar, Diana Saleiro, I. Caroline Le Poole

**Affiliations:** 1 Robert H. Lurie Comprehensive Cancer Center, Northwestern University, Chicago, IL 60611, USA; prathyaya.ramesh@northwestern.edu (P.R.); rohan.shivde@northwestern.edu (R.S.); dinesh.jaishankar@northwestern.edu (D.J.); diana.saleiro@northwestern.edu (D.S.); 2 Department of Dermatology, Northwestern University, Chicago, IL 60611, USA; 3 Division of Hematology-Oncology, Department of Medicine, Feinberg School of Medicine, Northwestern University, Chicago, IL 60611, USA; 4 Department of Microbiology and Immunology, Northwestern University at Chicago, Chicago, IL 60611, USA

**Keywords:** T cell, cytokines, tumor, effector function, immunotherapy, polyfunctionality, immune monitoring

## Abstract

**Simple Summary:**

Cytokines are molecules that help cells communicate at short range. Often, a single cytokine is measured in patient tissues to understand whether immune cells are actively clearing a tumor. We can learn whether a therapy works, even before changes in tumor burden are found. However, measuring multiple different cytokines better reflects ongoing anti-tumor activity. That is especially true for T cell-based therapies, as these cells secrete different cytokines when they eliminate tumors or not. We also need to consider that a single cytokine can perform activities that can either suppress, or support tumor growth. Taken together, we can best describe the function of T cells in a (pre) clinical setting by a palette of cytokines working together to paint a picture of ongoing immune responses to tumor cells.

**Abstract:**

Cytokines are key molecules within the tumor microenvironment (TME) that can be used as biomarkers to predict the magnitude of anti-tumor immune responses. During immune monitoring, it has been customary to predict outcomes based on the abundance of a single cytokine, in particular IFN-γ or TGF-β, as a readout of ongoing anti-cancer immunity. However, individual cytokines within the TME can exhibit dual opposing roles. For example, both IFN-γ and TGF-β have been associated with pro- and anti-tumor functions. Moreover, cytokines originating from different cellular sources influence the crosstalk between CD4^+^ and CD8^+^ T cells, while the array of cytokines expressed by T cells is also instrumental in defining the mechanisms of action and efficacy of treatments. Thus, it becomes increasingly clear that a reliable readout of ongoing immunity within the TME will have to include more than the measurement of a single cytokine. This review focuses on defining a panel of cytokines that could help to reliably predict and analyze the outcomes of T cell-based anti-tumor therapies.

## 1. Introduction

Cytokines are intercellular signaling molecules that control critical biological functions such as cell proliferation, differentiation, survival, cell cycle progression, and immune-cell activity [1]. Based on their function, cytokines can be divided into subgroups: lymphokines, monokines, chemokines, interleukins (IL), and interferons (IFNs) [2]. These secreted proteins act at short range as humoral regulators, attuning the functional processes of individual cells to maintain cellular homeostasis [1]. Importantly, cytokines are key mediators of anti-tumor immune responses by controlling activation of immune cells [2,3]. As such, the abundance and type of cytokines within the tumor microenvironment (TME) is an important biomarker to predict tumor progression [1]. However, further studies are required to fully elucidate the different biological functions induced by the presence of a single versus multiple cytokines, and by the “cross-talk” between cytokine-mediated signaling pathways [1].

Typically, the TME is characterized by presenting high levels of the anti-inflammatory, immunosuppressive cytokine IL-10, which facilitates tumor expansion [3]. Meanwhile, IFNs hold anti-tumor, anti-viral, and immunomodulatory properties [4], with IFN-gamma (IFN-γ) playing a critical role in defining a pro- versus anti-TME. IFN-γ has long been studied as the ultimate pro-inflammatory cytokine, with the responsibility to regulate anti-inflammatory responses. It also inhibits differentiation of immunosuppressive regulatory T cells (Tregs), and balances tissue destruction in chronic inflammation conditions [5,6]. Interestingly, depending on the biological context, IFN-γ can enhance or diminish the expression of tumor antigens, thereby determining the extent of anti-tumor immune responses [7]. Thus, some pro-tumor consequences of IFN-γ exposure exist, and its role may depend on the length of exposure and absolute abundance of the cytokine within the TME [5]. Additionally, while necessary, this cytokine does not seem to be sufficient on its own to halt the development of tumors [6].

Transforming growth factor-beta (TGF-β) operates in different capacities throughout disease progression. TGF-β can function as a tumor suppressor, inducing apoptosis and suppressing proliferation of pre-malignant and cancer cells [8]. However, as the name suggests, TGF-β can also mediate pro-metastatic and tumorigenic responses and support an immunosuppressive TME. TGF-β can help control inflammation and mediate immune tolerance [8]. The interchange of TGF-β with IFN-γ can be indicative of the effectiveness of cancer therapies and of an immune-active TME. However, the dual face of these cytokines is emblematic as to why a single cytokine is not satisfactory to adequately determine the ongoing immunity of the TME. Besides IL-10, TGF-β, and IFN-γ, it is the concerted action of multiple cytokines within the TME that mainly determines the extent and success of anti-tumor immune responses. Cytokines allow immune cells to transmit signals to each other and, under ideal conditions, promote a vigorous and harmonized response against target cells in both a paracrine and autocrine manner over short distances [9,10]. The anti-tumor activities of several cytokines have been established in animal models, serving as a premise for several cytokine-based immunotherapies [10]. Moreover, T cell engineering can be used for the expression of an antigen specific CAR or TCR to drive responses selectively towards tumor cells [11]. The pattern of cytokines expressed in response to immune cell therapy can then be used as a readout for anti-tumor control.

As important as cytokines are in predicting tumor progression and the success of cancer therapies, they can provide the identity and abundance of immune cells infiltrating the TME. Tumor infiltrating lymphocyte (TIL) populations include CD3^+^CD4^+^ and CD3^+^ CD8^+^ T cells, which can each be engaged in suppressing tumor growth [12,13]. B cells can also be present in the TME, and among them, regulatory B cells are known for the release of immunoregulatory cytokines that hinder anti-tumor responses by inhibiting natural killer (NK) cells and CD8^+^ cytotoxic T cells [14]. Inflammatory cells within the TME can either actively hinder tumor development or contribute to tumor progression [12]. More ambiguous roles have been assigned to T helper 2 (Th2) CD4^+^ T cell populations, which produce IL-4, IL-5, and IL-3 in support of B and T helper 17 (Th17) cell responses [15]. Thus, it will be imperative to expand our understanding of cytokine function to understand the true breadth and role that these biomarkers play in the activation of these immune cells and establishing anti-tumor immunity.

Identifying a defined palette of cytokines that can predict immune activation versus immunosuppression enables a better characterization of the TME. Here, we provide a magnified perspective of the cytokine patterns that represent the inner workings of immune cells, and specifically T cells responding within the TME. The observation of different secreted cytokine patterns can be associated with respective outcomes, and then used to accurately evaluate the effectiveness of immune-based therapies.

## 2. T Cell-Derived Cytokines as Biomarkers of Anti-Tumor Activity

**Figure 1:**
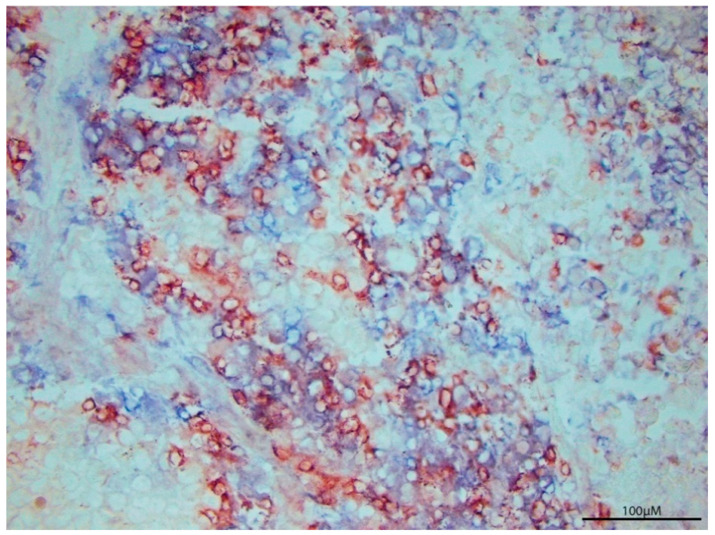
T cell Infiltration per se does not define tumor regression. P607 tumor tissue was surgically resected from a metastatic melanoma patient, revealing abundant T cell infiltration by immunoperoxidase labeled anti-CD8 (red) and alkaline phosphatase labeled anti-CD3 staining (blue). Cytokine analysis of tumor homogenates can reveal the prevailing immune response to better understand tumor expansion despite heavy T cell presence.

T cells are a notable indicator for characterizing ongoing immunity within the tumor microenvironment. However, T cell abundance is not an adequate marker of whether tumors will regress. Figure 1 showcases a human melanoma tumor with an abundance of T cells. This exemplifies the need to example T cell function over T cell abundance to understand the nature of tumor progression.

A combination of cytokine markers can provide reliable information on the capacity of T cells to act within the tumor. T cells are the primary source of each cytokine and these profiles are used to define T cell subsets [16]. Specific combinations of cytokines are associated with Th1, Th9, and Th17 T cells. As an additional parameter, most studies will include CD107A as a surrogate marker of cytotoxic activity.

In reference to the Th1 subset, the ratio of Th1 to Th2-associated cytokines provides information on the outcome of ongoing antitumor immune responses [17]. Representative cytokines for the Th1 subset, with classic anti-tumor activity include IFN-γ and IL-2 [18] and the Th1/Th2 ratio is critical for numerous immune responses [19]. Showing the ability of antigen specific Th1 cells through the use of adhesion molecules places the Th1 subset at the forefront of tumor rejection activity [17]. Th1 cells primarily stimulate CTL activation and differentiation and the secretion of cytokines such as IL-2, IL-12, IFN-γ, and TNF-α support CTL effector phenotype [20]. Additionally, Th1 therapy can induce lasting immunological memory to revive CTL generation upon tumor recurrence [17]. Critical to this work is the discovery that Th1 cells expressed adhesion molecules LFA-1/ICAM-1 [19], that allow transmigration into tumor tissues across tumor vessels, supporting tumor metastasis [17,19]. The cytokines secreted by Th1 T cells are commonly associated with a good prognosis for anti-tumor therapies [20]. Moreover, Th1 cytokines produced upon antigen stimulation directly induce the recruitment of effector cells such as CD8^+^ T cells, NKT cells, and NK cells to the tumor [17].

Th9 cytokines were designated as a separate T cell subset preferentially producing IL9 [18]. However, IL9 secretion per se is not restricted to Th9 cells, and can also be produced by Treg, Th17, or Th2 cells [18]. IRF4 expression combined with TGF-β and IL-4 exposure are critical for Th9 differentiation [18]. IL-9 is implicated in the anticancer effects of Th9 cells [18], whereas IL-2 supports the production of IL-9 [21]. The cytokine enhances dendritic cell survival and strengthens their capacity to induce and foster protective immunity [18]. Typically, Th9 cells produce low levels of IL-21, and also generate IL-25 and IL-1, but the anticancer effects of Th9 cells predominantly depend on IL-9 [18]. Originally assigned a function in combating parasitic infections, Th9 cells have been designated as anti-tumor T cells and their IL-9 expression can potentially induce bystander cell recruitment (such as NK cells and CD8 cells) [18]. Transcription factors, including STAT6, GATA3, PU.1, and IRF4, are central to Th9 polarization [18]. Of these, GATA3 and STAT6 have also been detected in Th2 cells. In other reports, IL-9 has been identified for targeted proliferation and activation of mast cells and enhancement of dendritic cell survival [18], highlighting IL-9 as a nominal marker for antitumor activity.

The Th17 cell subset does not exhibit stable differentiation and rather adorns a plasticity which allows it to adapt to its surroundings [22]. Th17 cells were identified as an individual subset based on their production of IL-17 [22]. Additionally, this subset produces IL-17F, IL-21, GM-CSF, and IL-22, whereas IL-23 is required for Th17 cell survival and pathogenesis [22]. Th17 cells have predominantly been associated with autoimmune and inflammatory diseases [22]. The plasticity of this subset places its role in cancer under debate, as Th17 can transdifferentiate into other subsets. Th1 conversion supports anti-tumor value, but Th17 can also morph into TR1, Th2, or TFH cells. Thus, Th17 are equipped to mediate several contradicting functions [22]. Within melanoma however, Th17 cells tend to spur protective immunity and establish tumor regression [22]. Within the TME, Th17 cells did not exhibit killing activity, but instead promoted dendritic cell and CD4^+^ and CD8^+^ T cell recruitment [22]. Th17 T cell function is thus difficult to classify due to its plasticity.

CD107a (also known as LAMP-1) is not a cytokine, but serves as a substitute marker of cytotoxic activity expressed by natural killer (NK), γδ T cells, and cytotoxic T cells, briefly found on the cell surface upon degranulation [23,24]. NK cells help mediate tumor cell clearance and viral infections [25]. Expression of CD107a is aligned with IFN-γ and TNF-α expression and cytotoxicity by T cells, and is likewise upregulated on activated NK cells, thus functioning as a general NK functional activity marker [25].

Besides T cell derived cytokines themselves, the cytokines that induce T cell activity can also be informative of the tumor microenvironment and can be used as treatments as well. Therapeutic cytokines for tumor patients can be subclassified into the IL-1, IL-2, and IL-12 families [26]. IL-1 is categorized as a pro-inflammatory cytokine and works as a T cell co-stimulator along with its role in inducing effector cell proliferation [26]. IL-2 was originally discovered as T cell growth factor and is key in promoting CD8^+^ mediated antitumor immunity [26], stimulating T cell growth, differentiation of effector T cells and Th1 cells, and activation of NK cells. In fact, it is the most used cytokine in cancer therapy [26]. Finally, IL-12 is capable of inducing Th1 and IFN-γ responses (activates JAK-STAT pathway after binding to its receptor) within T cells [26]. IL-12 is produced by dendritic cells and macrophages and like the other Th1 cytokines, plays a role in NK cell activation and Th1 differentiation [26]. IL-12 tends to display limited therapeutic potential, though IL-12-producing chimeric antigen receptor (CAR)-modified T cells potently destroy existing tumors [26].

Among T cell-derived biomarkers, Th1 cytokines IFN-γ, IL-2, and TNF-α present themselves as the most notable markers of responses to therapy [18,25]. The synergy of these and other cytokines (in a polyfunctional manner) as outlined in Table 1, can produce positive, potent, and efficient responses to cytokine therapies. Table 1 serves to highlight the most commonly measured cytokine responses across different tumor types with T cell-based therapeutics. By relating these secondary outcome measures to treatment outcomes, we create a foundation for a palette of cytokines that can better serve to relate therapeutic outcomes. From this table, we conclude that detecting a combination of IFN-γ, TNF-α and IL-2 is more informative than measuring a single cytokine such as IFN-γ alone. Moreover, the function of a cytokine within the tumor environment can be ambivalent and several factors can affect their in vivo functionality.

## 3. The Dual Face of IFN-γ and TGF-β

Of the many cytokines available, the balance between IFN-γ and TGF-β plays a pivotal role in determining the pro- versus anti-tumor characteristics and outcomes within the TME. However, simply measuring IFN-γ or TGF-β secretion in the TME is not enough to accurately characterize the anti-tumor potential of the TME. IFN-γ is a hallmark cytokine produced by Th1 cells, but also by CD8^+^ T cells, γδ T cells, NKT cells, NK cells, dendritic cells, and macrophages [6,33,34]. The biological functions exhibited by IFN-γ are derived from its interaction with the IFN-γ receptors IFNGR1 and IFNGR2 [33]. TGF-β is the hallmark cytokine involved in lymphoid organs and the immune system with a pivotal role in the maintenance of T-cell homeostasis [33]. TGF-β signaling is dependent on environmental cues within the TME and exhibits its dual role in response to these cues.

IFN-γ has long been prominently hailed as an anti-tumor environment-promoting cytokine [35]. IFN-γ affects both antitumor effector cells and tumor cells themselves [35]. This cytokine has a pleiotropic effect on several responsive cell types [6,33]. IFN-γ has direct anti-tumor effects by inhibiting tumor cell proliferation through induction of cyclin-dependent kinase inhibitor 1 (p21) expression and consequent cell cycle arrest [4,35]. Moreover, IFN-γ can upregulate expression of caspase-1, -3, and -8, promoting tumor cell apoptosis [4]. IFN-γ also stimulates an anti-tumor environment by increasing expression of classical MHC class I genes, cytotoxicity, effector functions, CD4^+^ and CD8^+^ T cell proliferation, and survival, while also decreasing Treg suppressive activity and endothelial cell proliferation [4,36,37]. The inhibition of tumor angiogenesis is another prominent function of IFN-γ in establishing an anti-tumor environment [36]. IFN-γ can inhibit angiogenesis directly in combination with TNF-α, or indirectly through the production of secondary anti-angiogenic molecules such as MIG and IP-10 [36]. However, IFN-γ can also promote angiogenesis by downregulating TNFSF15 [38].

Individual facets of IFN-γ activity are dependent upon factors such as signal intensity, signal duration, and other microenvironmental cues [4]. On one hand, tumor cells are a direct target of IFN-γ activity [39] and are also very vulnerable to destruction by IFN-γ activated immune effector cells [35]. Indeed, exposure to IFN-γ causes increased expression of IL-12, IL-18, and IL-17 by antigen presenting cell (APC), which encourages Th1 differentiation and supports CTL functions [37]. Further supporting its anti-tumor activity, a loss of responsiveness to IFN-γ signaling in tumor cells is associated with anti-apoptotic and proliferative activity [4,37]. On the other hand, chronic exposure to IFN-γ can mediate tumor immunoevasion [39] by the development and recruitment of myeloid-derived suppressor cells (MDSCs). Moreover, IFN-γ induces the expression of PD-L1 inhibiting T cell effector functions [4] promoting tumor progression and expression of MUC16 to promote tumor progression [4,35,39,40]. Furthermore, genomic instability induced by IFN-γ can cause phenotypic changes in tumor cells that lead to therapeutic resistance or support metastasis [39].

TGF-β is popularly categorized as a pro-tumor cytokine and plays an integral role in immunoregulation and cancer. It is instrumental in the promotion of differentiation and homeostasis of certain T cell populations [41,42]. This particular pleiotropic cytokine promotes an antitumor environment by increasing tumor cell apoptosis, reducing expression of growth factors and inhibiting cell cycle progression [41,43]. Conversely, unlike IFN-γ, high levels of TGF-β are typically considered a “negative prognostic indicator” [41]. Initially, TGF-β was simply defined as an immunosuppressant, but this cytokine has more than just immunosuppressive capabilities [41,42].

TGF-β inhibits differentiation of Th1 and Th2 cells but is required for Th17 cell differentiation [42]. TGF-β can also directly suppress CD8^+^ T cell responses in the TME by inhibiting expression of cytotoxic genes [42]. CD8^+^ T cells with impaired TGF-β signaling have been found to inhibit tumor development and evoke strong antitumor immune responses [42]. As a tumor suppressor, TGF-β inhibits tumor cell proliferation and induces pre-malignant cell apoptosis typically triggered within cells that suffer oncogenic stress [35].

The dual role of TGF-β is context-dependent as its downstream signaling effects vary depending on the progression of the disease [44]. During pre-malignant disease progression, TGF-β signaling promotes a pro-tumorigenic microenvironment [43]. As a tumor suppressor, TGF-β inhibits tumor cell proliferation and induces pre-malignant cell apoptosis typically triggered within cells that suffer oncogenic stress [35]. Furthermore, TGF-β can drive invasion, metastasis, and epithelial-to-mesenchymal transition (EMT) by influencing both the stroma and cancer cells [35]. When the disease progresses to late-stage cancer, the tumor is resistant to suppressive signaling effects due to TGF-β loss-of-function mutations [43].

As the disease advances, TGF-β functions as a crucial mechanism for immune evasion [8]. The protumor capacity of TGF-β relies largely on the tumor cells themselves. The ability of TGF-β to convert CD4^+^CD25^−^ T cells into iTreg cells is one of many methods to evade antitumor immunity [41]. Indeed, an increased presence of Tregs within the TME is driven by tumor-derived TGF-β [41]. Additionally, the conversion of dendritic cells into MDSCs occurs via tumor-cell produced TGF-β, which in turn stimulates Treg cell proliferation [41]. A TGF-β-abundant microenvironment thus facilitates the evasion of host immune surveillance [41].

High levels of IFN-γ are a known indicator of “good T-cells” but as indicated above, the role of IFN-γ in the TME is highly context-dependent [4]. In part, this context is provided by the temporal abundance of TGF-β. The ratio between these two cytokines is a more telling parameter to measure within the TME and these measurements can be further enhanced using cytokine analysis. However, the interplay of these dominant cytokines is not enough to accurately identify the status of tumor immunity within the microenvironment, mark T cell activity and response or determine tumor outcomes.

## 4. The Interplay of CD4^+^ and CD8^+^ T Cells Define Immune Responses in the TME

Therapeutic T cells within the TME can display varying levels of efficacy based on the cytokines they release, which ultimately defines the scope of functionality for that T cell. There is a mutually supportive relationship between CD4^+^ and CD8^+^ T cells; both cell types are components of the adaptive arm of the immune system and rely on antigen expression to initiate anti-tumor immunity [45]. CD4^+^ T cells are defined as either helper (Th) or suppressor T cells, which express unique transcription factors that direct the transcription of specific cytokine genes associated with each subset. CD4^+^ T cells most commonly associated with anti-tumor responses include Th1, Th9, and Th17 cells, which drive cell-mediated responses [46], while Th2 cells support the humoral arm of the adaptive immune response considered less effective at containing tumors [47]. In contrast, immunosuppressive CD4^+^ Tregs have an integral role in immune tolerance, thus actively interfering with anti-tumor responses [15,45]. Th22 T cells are associated with a poor prognosis [48] and are likewise known for interfering with ongoing anti-tumor immunity [49]. However, the precise role of IL-22 producing Th22 cells is poorly defined [50].

**Figure 2:**
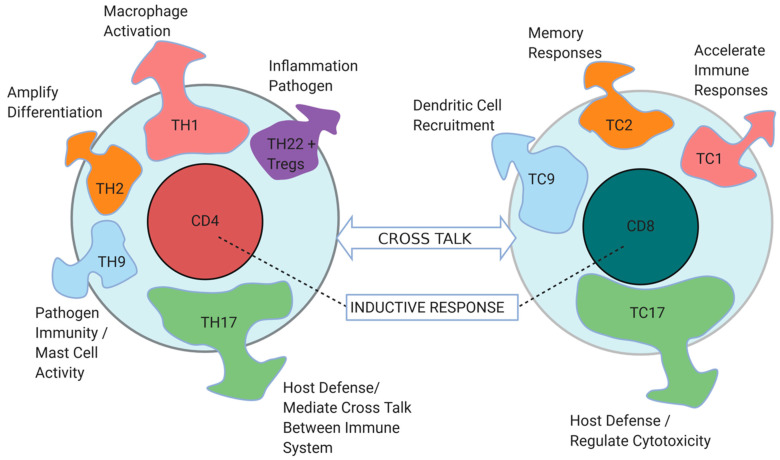
CD4^+^ and CD8^+^ T cell identity and functions are regulated by a palette of cytokines. CD4^+^ and CD8^+^ T cells interact within the tumor microenvironment and secrete different sets of cytokines, which control several and distinct immune responses, represented by different colors in the diagram. Cytokines secreted by each T cell type are as follows: IFN-γ, IL-2, TNF-α, and TNF-β by TH1, IL-4 and IL-10 by Th2, IL-9 by TH9, IL-17, IL-17F, IL-21, and IL-22 by Th17, IFN-γ and TNF-α by TC1, IL-4 and IL-12 byTC2, IL-9 by TC9, and IL-17A, IFN-γ, TNF-α, IL-21, and IL-22 by TC17. Arrow thickness and size represent the magnitude of influence that a specific subset of cytokines have on the tumor microenvironment immune responses. CD8^+^ T cell functions are regulated by CD4^+^ T cells and the cross talk between the two cell types is critical for establishing an effective anti-tumor immunity and microenvironment. Figure created with BioRender.com.

CD8^+^ T cells, on the other hand, are the cytotoxic subset of T cells that have traditionally been pursued as the most critical for tumor control. And yet, as depicted in Figure 2, ongoing crosstalk between CD4^+^ Th1 and CD8^+^ T cells is an often-overlooked requirement for immune activation and is necessary for suppressing tumor growth [45]. Th1 cells promote the ability of CD8^+^ T cells to produce IFN-γ and IL-2, and conduct clonal expansion [15,51]. Moreover, some CD4^+^ T cells display cytotoxic functions of their own [47]. Tumor-reactive CD4^+^ cells are adept at communicating with diverse innate and adaptive immune cell types within the TME [45]. CD4^+^ cells are effective educators and inducers of other cell types. For example, CD4^+^ T cells can license dendritic cells to activate CD8^+^ cytotoxic T cells, or induce B cells (Th2) to activate a switch between different classes of antibodies [52]. Th9 T cells can support mast cell activation and cytotoxic activity [53]. Th17 cells can affect endothelial cell function and vascular integrity, while presenting a plastic population that can emerge as Th1 cells [54]. As such, CD4^+^ T cells are oftentimes responsible for the cytokine composition found in regressing tumors. Al- though CD4^+^ T cells generally display minimal in vivo or in vitro lytic activity against tumors, one could say that their activation in the TME determines tumor rejection to a greater extent than activation of CD8^+^ T cells [52,55].

CD8^+^ T effector cells are categorized into two main classes: CD8^+^ type 1 (Tc1) cells, which primarily produce IFN-γ and TNF-α, and type 2 (Tc2) cells, which produce IL-4, IL-5, IL-10, and IL-13 [36]. Tc1 cells have the potency to kill target antigen bearing cells, via the release of perforin and granzyme B [56]. These cytotoxic mediators are released into the immunological synapse, whereupon T cells secrete cytokines such as IFN-γ and TNF-α to further induce the immune response [57]. On the other hand, Tc2 cells often aggravate diseases and can only mediate low levels of cytotoxicity [57]. Thus, type 1 cytokines are associated with potent cytotoxicity and establish lasting anti-tumor immunity.

Tumor immunotherapy is evolving to include antigen specific, transgenic T cells [58]. In areas of cancer immunotherapy where traditional therapies have proved unsuccessful, a different approach is required; namely, adoptive T cell transfer [58]. CD8^+^ T cells have become the front runner in several cancer immunotherapies, given their cytolytic activity [56]. Besides adoptive transfer of TCR transgenic CD8^+^ cytotoxic T cells responding to a tumor-associated antigen, the concept of chimeric antigen receptor-transduced T cells has taken flight [56]. One difference among these 2 approaches is their dependence (or independence) on a CD4^+^ or CD8^+^ co-receptor to function [59]. Indeed, chimeric antigen receptor (CAR) transduced CD4^+^ (CAR4) and CD8^+^ (CAR8) T cell subsets each respond to antigen, with individually defined roles in the anti-tumor response [60].

The relative efficacy of CD4^+^ and CD8^+^ T cells can be observed in several models. For example, in an attempt to define the consequences of TCR activation of CAR T cell potency, researchers designed a comparative murine study which resulted in notably different responses amongst CAR4 and CAR8 cells [61]. T cells exhibited increased signs of exhaustion in the presence of the TCR marker with an increase in PD-1 and LAG3 expression, in contrast to CAR4 cells, inhibiting their ability for leukemia clearance [61]. In this study, parallel activation of the existing TCR and the newly introduced CAR obliterated CAR8 efficacy within the TME [61]. The CAR8 T cells were more vulnerable to apoptosis and expressed T cell markers which signal a diminished ability to clear the targeted B cells [61]. On the other hand, CAR4 T cells displayed resistance to immunosuppression following TCR stimulation, specifically to early activity and expansion. This may be due to copious recognition of tumor antigen presented in the context to MHC class I (HY and OVA peptide specific mice) and subsequent TCR-mediated activation of CD8^+^ cells, while CD4^+^ T cells are TCR-activated only by antigen presenting cells that carry MHC class II molecules [13,61]. Moreover, CD8^+^ T cells are more restricted in their choice of antigenic peptides to respond to, with very little allowance for changes to the cognate peptide [62].

In summary, when comparing the contribution between CD4^+^ and CD8^+^ T cells against tumors, CD4^+^ T cells can be assigned a mentoring and directive role [45]. The effectiveness of T cells and their responses is significantly enhanced when multiple cytokines are secreted into the TME. In correlation with Figure 2, the cytokines most telling of favorable responses are IFN-γ (Th1, TC1, TC17), TNF-α (Th1, TC1, and TC17), and IL-2 (Th1), as shown in Table 1 studies 1,2,5, and 6. Current therapeutics can be associated with adverse events that are also marked by cytokines [63]. This combination of secreted cytokines can be highly instrumental in understanding anti-tumor functionality.

## 5. T Cell Polyfunctionality

T cell subset-associated transcription factors are considered the primary indicators for T cell classification [64]. However, T cell classification usually relies on the expression of a combination of cell surface glycoproteins by flow cytometry analyses [27]. This array of surface molecules aligns with specific functional features, which allow classification of T cells according to their activation status, including the identification of the cytokines that they produce. The ability to produce multiple cytokines is dependent on the TCR binding affinity and antigen-sensitivity of a T cell [29]. Polyfunctionality, the ability of T cells to produce multiple cytokines, enhances immune responses to infectious diseases and following vaccinations [27,29]. However, the process by which polyfunctional T cells arise to enhance immune response is not yet fully understood [27].

A first step towards answering this question includes single-cell analysis of cytokines secreted by polyclonally activated peripheral blood human CD8^+^ T cells [27]. Here, dynamic single-cell analysis of cytokine release by individual T cells was determined over time by enzyme-linked immunosorbent assay (ELISA) (Table 1, study #1). This study revealed the presence of dynamically evolving T cell polyfunctional responses, suggesting that T cells present functional plasticity over time. The initial cytokine pattern in response to antigen stimulation is essentially monofunctional, whereas polyfunctionality develops later and represents a transient functional state [27]. Among the cytokines analyzed, secretion of IFN-γ or IL-2 showed remarkable consistency and dominance among individually defined polyfunctional states and secretion was markedly increased over time, while cells secreting TNF-α diminished [27].

In another study [28] (Table 1, study #2), different peripheral blood T lymphocyte subsets and renal parenchymal tissue were analyzed to better understand the relationship between immune cells and cancer cells. A notable observation was the increased production of IL-10 amongst the CD4^+^ and CD8^+^ tumor infiltrating T lymphocytes, but this response was not observed among autologous PBMC’s from patients responding to therapy [28]. Through immunohistochemistry, both CD8^+^ effector memory cells and polyfunctional CD4^+^ T cells were observed in abundance among tumor infiltrating lymphocytes (TILs) [28]. While intra-tumoral CD8^+^ T cells were observed, CD4^+^ T cells were the predominant cell type in TILs of the renal tumor and constitute the more functional and experienced effector memory cells [28].

Further, in a study to evaluate the effects of anti-PD-1 therapy in melanoma patients, polyfunctional and persistent, older CD8^+^T cells were found to drive polyfunctionality of newer monofunctional CD8^+^ T cells over time (Table 1, study #3) [29]. CD107a expression among tumor reactive CD8^+^ T cells indicates that an accumulation of polyfunctional T cells occurs in their peripheral pool [29]. This accumulation serves as a predictor of lasting immune responses. Polyfunctional T cells were consistently present for up to one year post-infusion and changes in non-tumor reactive CD8^+^ T cells are likely indicative of a replacement by the infused de novo population [29]. Indeed, expression of exhaustion marker PD-1 in T cells expressing both IFN-γ and TNF was associated with long-term memory responses.

The presence of polyfunctional T cells has also been used as a biomarker of success for immunotherapeutic strategies used in glioblastoma (GBM) patients (Table 1, study #4) [30]. Injection of cytomegalovirus (CMV) antigens in conjunction with dendritic cells was used to enhance polyfunctionality among CMV-reactive T cells in GBM patients, and consequently enhance immune anti-tumor responses. Blood sample analyses established that circulating patient T cells are not actively polyfunctional until ex vivo exposure to the CMV antigen. Vaccinated patients displayed improved survival, correlating with increased polyfunctional T cell abundance [30].

In another study, cytokine treatments were used to induce T cell polyfunctionality profiles (Table 1, study #5) [31]. IL-2 treatment combined with anti-CD3 therapy was found to restrict tumor cell proliferation and enhance secretion of TNF-α. Where additions of single cytokines did not induce significant changes in profile, a combination of exogenous cytokines (IL-2+, IL-12+ IL-21 or IL-2+, IL-12, IL-18) was needed for polyfunctionality induction and treatment efficacy resulting in a marked increase of IFN-γ, TNF-α, and CCL4 expression [31]. Researchers used a Boolean gate analysis to monitor changes in T cell polyfunctional permutations. The overall observations apply to both CD8^+^ and CD4^+^ T cell subsets, though relative to proliferation, cytokine combinations were required primarily for CD8^+^ T cells.

In study #6 (Table 1) the need for polyfunctionality to support successful TIL therapy is definitively demonstrated [32]. Polyfunctionality was observed in TILs, which secreted IL-2 and at least one of the other main cytokines, IFN-γ or TNF-α. Overall, the results show that the presence of polyfunctional, tumor reactive CD4^+^ T cells can be a desirable prognostic biomarker for survival [32].

The role of cytokines for T cell differentiation, migration, survival, transformation, and replication has also been evaluated in vivo [1]. Polyfunctional CD4^+^ T effector cells were found to mediate dendritic cell licensing and enhance CD8^+^ T cell activation within the TME [65], contributing to robust antitumor immune responses and preventing B-cell lymphoma recurrence in vivo. CD4+ cells can act upon the dual secretion effects of IFN-γ and TNF-α by inhibiting tumor angiogenesis [65].

When CD8^+^ T cells from the same host were transduced to express three different TCRs (SILv44, R6C12, and T4H2) responding to the same HLA-A2-restricted peptide, we found that the resulting cytokine responses varied, suggesting that subtle changes in TCR structure can affect the resulting cytokine secretion patterns of host T cells and impact anti-tumor responses in ways that do not correlate with IFN-γ secretion, but rather with IL-17 expression levels [66].

Together these studies add to our understanding of polyfunctionality enhancing T cell function. The release of multiple cytokines and their synergistic capabilities significantly increases treatment efficacy and can also exogenously enhance T cell function in an autocrine fashion while supporting anti-tumor immunity within the TME. In order to accurately gain an understanding of how and when these cytokines are released, the use of reliable and informative analysis techniques is necessary.

## 6. Monitoring Cytokine Abundance

Recent technological advances have improved our ability to detect multiple cytokines and assign cytokine production to respective immune cells. Techniques such as FACS, ELISA, and ELISpot, are fundamental assessment tools that provide basic information about cytokine production [67,68]. More recently developed technologies offer greater signal separation to evaluate for a greater number of analytes and can provide additional single cell readouts of cytokine production, location, or function over time [69,70,71].

Flow cytometry or fluorescence activated cell scanning (FACS) is a commonly used technique that can provide quantitative information by intracellular cytokine staining (ICS) on a per cell basis [72,73]. ICS is a hallmark method for visualization of cellular responses and in particular, T cell responses [73]. However, this technique lacks the sensitivity to detect cytokines at low levels [68]. Flow cytometry does not determine where the cytokine is being released, nor whether the artificial backup created by chemically preventing secretion influences downstream production of the cytokine [73]. CyTOF (mass cytometry) is a recently developed technique, which adopts the format of flow cytometry (FC) using the precision of mass spectrometry [74], and holds the ability to survey millions of cells per sample for over 40 cellular parameters in parallel at the single cell level [74,75]. CyTEK offers a more accessible alternative to this technique, using spectral analysis to separate fluorochromes that are close in maximum emission wavelengths [76].

Another sensitive technique for cytokine detection is quantitative polymerase chain reaction (qPCR), which allows detection of cytokines being actively transcribed. This is a targeted approach requiring specific probes to detect cytokines transcripts from bulk RNA. With the advent of next generation sequencing (NGS) technologies like ribonucleic acid (RNA) sequencing (RNA-seq), unbiased, multi-target analysis can be performed and rare cytokine transcripts can be detected. [77,78]. Bulk RNA analysis holds shortcomings such as an inability to identify the source of the gene, and to reveal whether actual protein is made and secreted, and lacks delivery of a holistic analysis to the TME or cytokine secretion [79].

Enzyme-linked immunosorbent assay (ELISA) is yet one more analytical method to determine the total concentration of a secreted cytokine present in biological fluids or tissue culture supernatants [72,79]. However, this bulk cytokine estimation method does not provide information at a single-cell level [80,81]. Enzyme-linked immunospot assays (ELISPOTs) do report primarily qualitative data about cytokine-producing cells, enabling data regarding the number of activated cells and multiple secreted cytokines at the single cell level [68].

Analytical platforms for multiplexed cytokine detection such as Luminex and Meso Scale Discovery (MSD) are highly sensitive and use significantly less sample compared to a traditional ELISA [82]. Luminex is a bead-based immunoassay wherein fluorescent beads are coated with capture antibodies to specific analytes and are incubated with the samples [83]. MSD immunoassays are an advanced sandwich-based ELISA wherein analytes are detected using sulfo-tag conjugated detection antibodies instead of fluorescent-conjugated antibodies [84]. Both these assays, however, do not enable the phenotyping of cytokine secreting cells and are intended for fluids analysis.

More recent analytical tools seek to bridge the gap between the breadth of information captured, and the precision of that information. For example, IsoLight can be used to determine functional single cell secretion of multiple cytokines [85]. The IsoCode chip allows for the combined technology of a sandwich ELISA, fluorescence signal detection, and a quantitative measurement protein secreted per cell [85]. The Berkeley Light Switch operates similarly, however, the cells are moved to individual wells by light, so that all cells are analyzable [71]. Visium technology from 10× Genomics combines transcriptome probe field with tissue histology to provide the benefits of localized transcriptome analysis, allowing for combined information about tissue location at the protein level, and transcript quantification at the RNA level [86].

In summary, a variety of technologies and platforms are available to detect cytokines secreted within the TME. The choice of technology depends mostly on the type of analyses to be done and the cost. Table 2 places the emphasis on a single, popular therapeutic, (CD19 CAR) to highlight that a colorful palette of cytokines provides granularity and functional insight into therapeutic outcomes. This table outlines clinical trial studies in which different cytokine monitoring techniques were used and illustrates how cytokine detection is useful to evaluate anti-tumor responses.

## 7. Conclusions

The detection of cytokines and cytokine based anti-tumor immunotherapies, particularly polyfunctional T cell-based therapies, has shown promise in regenerating immune responses within the TME. Cytokine detection can prove a powerful tool to tailor immunotherapies in the fight against cancer. By identifying the unique combination of cytokines that are secreted as a positive response to respective cancer therapies, researchers can advance the methods by which they target and attack tumors. Further studies to explore and identify the ideal palette of cytokines, using of single cell cytokine monitoring techniques can increase the precision with which we characterize the ongoing immunity within the TME and can re-design the manner in which we establish anti-tumor immunity.

## Figures and Tables

**Table 1:** Correlating cytokine profiles and T cell responses.

Study	Cytokines Probed and Monitoring Methods	Patients/Source of T Cells	Cancer Type and Treatment	T cell Treatment Outcome Response and Correlated Cytokine
#1 [27]	IFN-γ, IL-2, TNF-α FACS analysis, ICS, ELISpot	Healthy donors	CD3^+^ T cells isolated from peripheral blood	IL-2 (mostly among CD8^−^ T cells) and IFN-γ secreting cells increased, TNF-α secreting cells decreased. *IFN-γ and IL-2 secreting cytokines showed functional state persistence.*
#2 [28]	TNF-α IFN-γ, IL-10, IL-17, IL-2 Intracellular cytokine staining of CD4^+^ and CD8^+^ T cells, in renal parenchyma tissues	Peripheral blood, fresh tumor, and autologous renal parenchyma	Renal cell carcinoma PBMC and TIL thawed and analyzed for cytokine release.	IL-10 increased among CD4^+^ and CD8^+^ subsets; TNF-α (CD4^+^ and CD8^+^), IFN-γ (CD8^+^) increased after activation. Some patients had increased IL-17 in CD4^+^ TIL. CD107a surface expression found in CD8^+^ and some CD4^+^ cells post- activation. *Cytokine secretion pattern of responders: TNF-α, IFN-γ, IL-2 with little IL-5.*
#3 [29]	TNF, IFN-γ, CD107a (cytotoxicity marker). FACS analysis, cytotoxicity assays, phenotype analysis, flow cytometry	Serial blood sample obtained from TIL treated patients	Melanoma IL-2 based TIL therapy	CD8^+^ T cells expressing CD107a were fewer than cytokine producing cells. *Most CD107a + cells also produced one cytokine.*
#4 [30]	IFN-γ, TNF-α, CCL3 IFNγ ELISPOT assay, FlowJo	22 CMV seropositive patients	Glioblastoma In vitro generation of (CMV) pp65 T cells and CMV pp65- DCs from PBMCs	Patients receiving CMV pp65 T cells had more IFNγ^+^, TNFα^+^ CCL3^+^ pp65 specific CD8^+^ T cells. *Survival in treated patients correlated with expression of IFNγ, TNFα and CCL3.*
#5 [31].	IFN-γ^2^,TNF-α^1,2^, IL-2, IL-12, IL-18, IL-21 CCL4^1,2^, CD107a^2^(cytotoxicity marker) Flow cytometry, ELISA, Bio-Plex	Bulk ascites cell preparations from high-grade serous EOC patients	Epithelial ovarian cancer (EOC) Exogenous cytokine therapy and introduction of EOC ascites environment on T—cell polyfunctionality	IL-1^+^, IL-12^+^ IL-18 enhanced IFNγ (by CD8^+^ cells), TNF-α, and CCL4 expression *Cytokine combination synergistically induced polyfunctional responses and decreased cytokine negative or monofunctional T cells.*
#6 [32]	IFN-γ^3^, TNF-α^3^ IL-2^3^ Flow cytometry, immunohistochemistry	25 treatment- naïve NSCLC patients with clinical stage I-Iva tumors.	Non-small cell lung cancers (NSCLC) TIL therapy	CD4^+^/CD8^+^ cells producing either 2 or 3 of the cytokines were most informative. *TNFα and IL-2 were crucial to T cell mediated immunity.*

**Table 2:** Correlation between cytokine detection and treatment outcomes in clinical trials using CD19- targeted CAR-modified T cells.

Study	Patient/Sample Measured	Cytokines Probed	Monitoring Techniques	Patient Outcomes	Correlation between Cytokines Detected and CAR T Cell Function
Study 1: NCT02963038 [87]	10 patients Serum concentration	Il-1β, IFN-α2, IFN-γ, TNF-α, MCP-1, IL-6, IL-8, IL-10, IL-12p70, IL-17A, Il-18A, IL-18, IL-23, IL-33	Flow cytometry, qPCR	80% achieved MRD 30% have remained in remission state. 10% achieved complete remission. Long-term engrafted CAR-T cell clone CD19 activity observed in one patient for >2 years. 40% experienced grade 2 or higher CRS.	High concentrations of IFN-γ, IL-6, IL-8, IL-18, and MCP-1 correlate with CAR-T cell expansion.
Study 2: NCT01044069 [88]	53 patients Serial serum samples	IFN-γ, IL-6, IL-10, IL-15, TNF-α	Luminex FlexMAP 3D system, 38-plex cytokine detection assays	83% achieved complete remission 42% experienced infections.	Cytokine Release Syndrome (CRS) secretion of: IFN-γ: expressed by a greater # of patients w/o infection. IL-6: Patients with CRS grade 2 and 3 had more infections than without. IL-10: expressed by a greater # of patients without infection. Il-15: Patients with CRS grade 3 had slightly more infections than without. TNF-α: Patients with grades 4-5 CRS had more infections than without.
Study 3: NCT01626495 [89]	50 patients Serum concentration levels	43 cytokines tested (not individually listed)	Luminex bead array, FlexMap 3D system	98% saw B-cell ALL with CD19 expression Neurotoxicity observed in 46% patients.	Serum IL-2, IL-15, IL-4, and HGF concentrations were notably higher in patients with neurotoxicity. TNFR-1 significantly higher in patients with encephalopathy. 22 cytokines accurately predict neurotoxicity. Predicting regression: IL-12, sgp130, sRAGE, sTNFR-1, sVEGFR, and sVEGFR2.
Study 4: NCT01865617 [90]	47 patients Serum concentrations	IL-7, IL-15	qPCR, Luminex Assay	Objective response observed in 51% of patients. 40% achieved complete remission CRS grade 1–3 observed in a subset of patients	High levels of IL-7 correlate with favorable outcomes. IL-7 concentration increases along with serum IL-15 levels.
Study 5: NCT00924326. [91]	22 patients Coculture and Serum concentrations	32 total cytokines: Granzyme B, IFN-γ, MIP-1α, perforin, TNF-α, TNF-β,IL-2, IL-5, IL-7, IL-8, IL-9, IL-12, IL-15, IL-21 IL-2, IL-10, IL-13, IL-22, TGF-β1, IL-1B, IL-6, IL-17A, IL-17F, MCP-1, MCP-4, CCL-11, IP-10, MIP-1β, sCD137, sCD40L, RANTES	PCR, MULTI-SPOT, EMD Millipore Luminex xMAP multiplex assays.	70% objective response to CAR-T cell therapy. 65% observable CRS of grade 3 or higher.	Both polyfunctional CD4^+^ and CD8^+^ T cells secrete: IFN-γ, IL-8, IL-5, granzyme B, and/or MIP-1α. CD4+ population contained IL-17A^+^ polyfunctional T cells. Responders had higher levels of inflammatory, regulatory, chemoattractive, stimulatory, and effector cytokines).
Study 6: NCT00924326. [92]	8 patients Serum concentration	IFN-γ, TNF, IL-2, CD107a (cytotoxicity marker)	ELISA, ICS followed by flow cytometry detection, CD107a degranulation assay. PCR	75% attained remission. 50% had prominently elevated serum levels of IFN-γ and TNF	CAR T cells were the source of inflammatory cytokines IFN-γ and TNF found in some patient sera.
Study 7: NCT01865617 [93]	37 patients: relapsed or refractory CD19^+^ NHL Serum concentrations	IFN-γ IL-6, IL-8, IL-10, IL-15, TGF-β	Luminex Assay	89% receiving CAR T cell infusion saw objective response. Severe CRS observed in 4/32 patients post Cy/Flu conditioning. Severe neurotoxicity observed in 9/32 patients.	Cy/Flu conditioning induced higher response rates. Peak serum concentrations for IL-6, and IFN-γ observed in correlation with sCRS and Cy/Flu conditioning. Patients with grade ≥ 3 CRS saw increased serum concentrations of IL-6, IFN-γ, IL-15, IL-2, IL-18, and reduced TGF-β. High levels of IL-6, IFN-γ, IL-15, IL- 8, and IL-10 and low levels of TGF-β correlated with severe neurotoxicity.
